# Sequential therapy with darolutamide in patients with non‐metastatic castration‐resistant prostate cancer resistant to enzalutamide or apalutamide

**DOI:** 10.1002/cam4.5189

**Published:** 2022-08-31

**Authors:** Saizo Fujmoto, Kazutoshi Fujita, Mitsuhisa Nishimoto, Mamoru Hamaguchi, Ken Kuwahara, Mamoru Hashimoto, Shogo Adomi, Takafumi Minami, Masahiro Nozawa, Kazuhiro Yoshimura, Hirotsugu Uemura

**Affiliations:** ^1^ Department of Urology Kindai University Faculty of Medicine Osakasayama Osaka Japan; ^2^ Department of Urology, Mimihara General Hospital, Sakai Osaka Japan

**Keywords:** apalutamide, ARAT, darolutamide, enzalutamide, nmCRPC, prostate cancer

## Abstract

Enzalutamide, apalutamide, and darolutamide are currently recommended for patients with non‐metastatic castration‐resistant prostate cancer (nmCRPC), but cross‐resistance of androgen receptor‐axis‐targeted therapies (ARAT) occurs. Because darolutamide has a distinct chemical structure to other non‐steroidal antiandrogens, it may be effective for nmCRPC patients resistant to enzalutamide or apalutamide. We retrospectively evaluated the efficacy of switching to darolutamide in patients with nmCRPC. We included nine nmCRPC patients who experienced biochemical progression on enzalutamide or apalutamide and were switched over to darolutamide. Five patients (55.5%) had a PSA response >50% decline after starting darolutamide, with an average of 73% PSA decline. Median progression‐free survival was 6 months. In conclusion, an ARAT switch from enzalutamide or apalutamide to darolutamide might be effective for nmCRPC. Although the validation in a large‐scale cohort is necessary, the switch to darolutamide could be a promising therapeutic option after the progression of 1st line ARAT in nmCRPC patients.

## Graphical abstract

In this study, we retrospectively evaluated the efficacy of an androgen receptor‐axis‐targeted therapies (ARAT) switch from apalutamide or enzalutamide to darolutamide for non‐metastatic castration‐resistant prostate cancer (nmCRPC). More than half of the patients had a PSA response of more than 50% decline after starting darolutamide. Darolutamide could be the option of sequential therapy in patients with nmCRPC resistant to other ARAT.

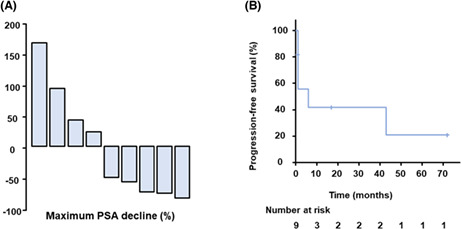

Non‐metastatic castration‐resistant prostate cancer (nmCRPC) is defined as increasing prostate‐specific antigen (PSA) in the setting of castrate testosterone levels and no detectable metastases by conventional imaging. nmCRPC is a poor prognosis, and aggressive treatment is necessary to prolong survival. The SPARTAN, PROSPER, and ARAMIS phase III trials demonstrated improved overall survival in patients with nmCRPC by treated with enzalutamide, apalutamide, and darolutamide, respectively.^1^

However, there is no consensus for treating patients with elevated PSA during an androgen receptor‐axis‐targeted therapies (ARAT) with no evidence of distant metastasis in imaging studies. ARAT switch becomes an option in such cases, but cross‐resistance exists between these agents, in particular between enzalutamide and apalutamide, due to their similar chemical structure. This becomes problematic making it difficult to anticipate the efficacy of switching between these agents. In contrast, the chemical structure of darolutamide is different from that of the other two agents. Thus, darolutamide may be effective as an ARAT switch.^2^ In this case series, we retrospectively analyzed the efficacy of ARAT switching to darolutamide in patients with nmCRPC who were resistant to enzalutamide or apalutamide. Computed tomography, magnetic resonance imaging, and bone scintigraphy assessed the presence of metastases. Kaplan–Meier curves analysis was performed for progression‐free survival. PSA doubling time (PSADT) was calculated from the time of nadir after the first hormone therapy until the diagnosis of CRPC. PSA response was defined as a decline of at least 50% from baseline in the PSA level. Statistical analysis was performed using R (version 4.1.2).

The study included nine patients with nmCRPC who had previously received other ARATs and took darolutamide between January 2020 and December 2021. The ethics committee of the Faculty of Medicine, Kindai University, approved the study protocol (approval number: R02‐247) and waived individual consent for this retrospective analysis. Patients' characteristics are summarized in Table 1. At the start of darolutamide treatment, median serum PSA levels were 3.1 ng/ml (range 0.13–20.73). Enzalutamide was used in six patients and apalutamide in eight patients as prior therapy for nmCRPC. Five patients received both of enzalutamide and apalutamide, which they developed resistant to. Five patients (55.5%) had a PSA response after starting darolutamide, with an average of 73% PSA decline (Figure 1A). The median progression‐free survival was 6 months (95% CI. 0.1090, 0.708) (Figure 1B). The nine patients included one patient in which the patient was switched from apalutamide to darolutamide due to an adverse event, but there was no PSA response in that patient. When comparing characteristics between responders and non‐responders, there were statistically no differences in age, Gleason score, PSA levels, clinical stage at diagnosis, history of local therapy, PSADT, or PSA levels at the start of darolutamide.

**TABLE 1:** Patient characteristics

Characteristics of the patients (*n* = 9)	
Age (years, median [range])	75 (71–87)
PSA doubling time (months, median [range])	3.8 (1.7–14.2)
Gleason score (7/8/9/10/unknown)	1/2/2/3/1
N stage (0/1/unknown)	5/1/3
Prior treatment (*n* [%])	
Enzalutamide	6 (66.7%)
Apalutamide	8 (88.9%)
Prior local therapy	4 (44.5%)

Abbreviation: PSA, prostate‐specific antigen.

**FIGURE 1:**
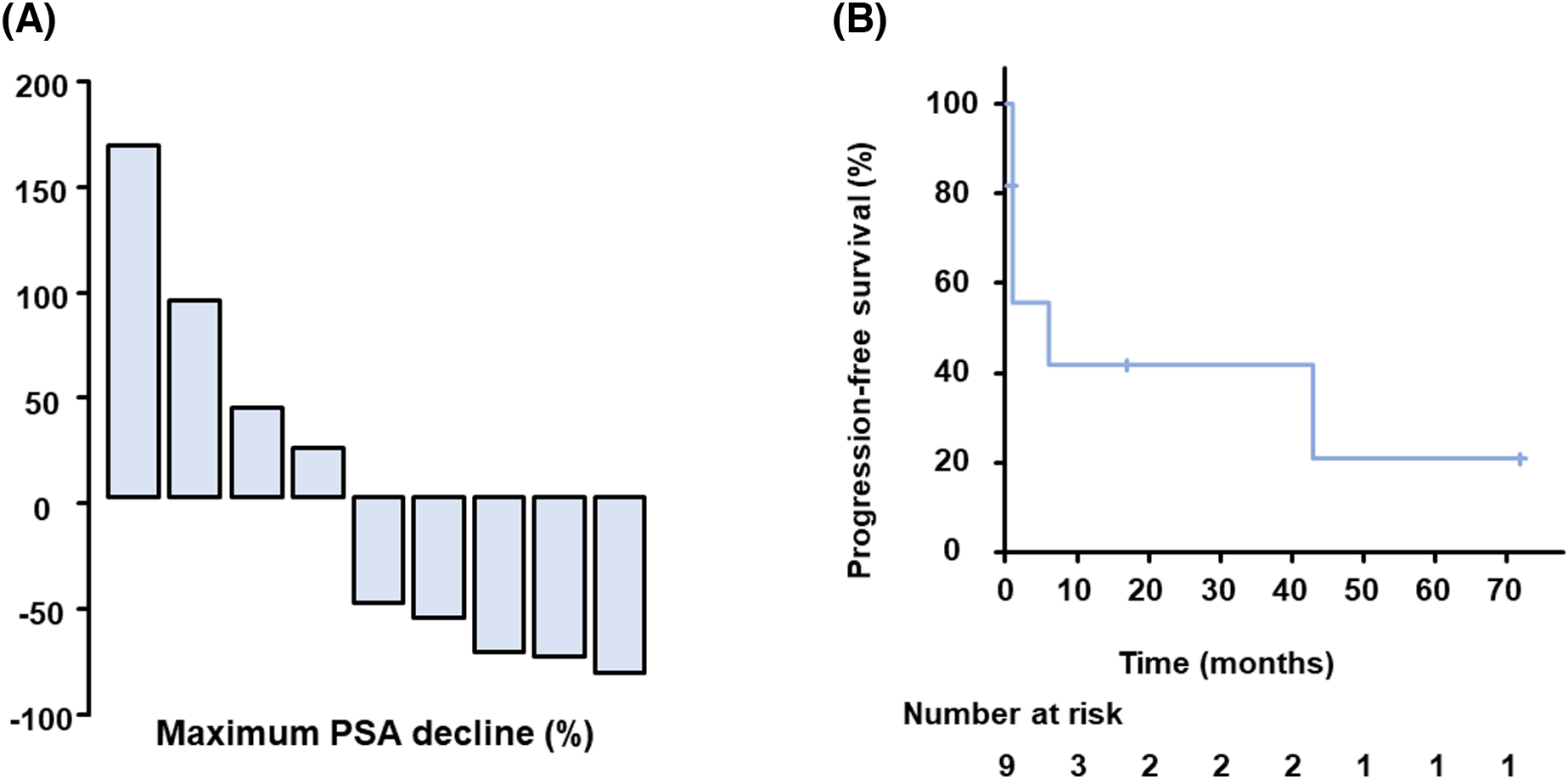
(A) Waterfall plots showing the response indicated by changes in PSA levels from the baseline in nmCRPC patients switched over to darolutamide. (B) PSA progression‐free survival in nmCRPC patients switched over to darolutamide. PSA, prostate‐specific antigen.

Regarding survival benefits for nmCRPC, there is little difference between enzalutamide, apalutamide, and darolutamide, as shown by SPARTAN, PROSPER, and ARAMIS trials.^1^ Apalutamide was developed from enzalutamide as a lead compound and had a similar chemical and steric structure. On the other hand, darolutamide has characteristics different from the other two agents, such as hydroxyl groups attached to the pyrazole ringside chain and a linear system in the center of the structure, which may have contributed to the efficacy of the switching.^3^ Furthermore, a network meta‐analysis showed that darolutamide has a higher overall survival rate and a better safety profile than enzalutamide and apalutamide.^4^

AR mutations in response to antiandrogen therapy are one of the significant factors implicated in acquiring drug resistance to ARATs in prostate cancer. Mutations arising in AR activate the receptor with drugs or non‐specific ligands, thus promoting cancer progression.^5^ Recent studies have reported that darolutamide inhibits the activity of more AR mutants than other ARATs. For example, one experiment evaluated the in vitro response of 68 AR mutants identified from CRPC patients to drugs. Bicalutamide and enzalutamide showed complete or partial activation in 43 and eight mutants, respectively. In contrast, darolutamide showed activation in only one mutant.^6^ This high activity of inhibition of AR mutants by darolutamide may have contributed to the response after the progression by enzalutamide or apalutamide.

The ARAT switch to abiraterone from enzalutamide is effective in some metastatic castration‐resistant prostate cancer patients.^7^ Abiraterone is a CYP17A1 inhibitor, a separate drug class from other ARATs due to differences in the mechanism of action. Like abiraterone, we expect that darolutamide may be effective as an ARAT switch due to the difference in chemical structure. Moreover, it is possible that switching to apalutamide and enzalutamide may be also effective in nmCRPC patients who have developed resistance to darolutamide.

Darolutamide was effective as sequential therapy for more than half of nmCRPC patients resistant to enzalutamide or apalutamide in this case series. Apalutamide, enzalutamide, and darolutamide are recommended options for patients with nmCRPC, if PSADT is less than or equal to 10 months. Progression to the metastatic disease would worsen the survival in patients with prostate cancer. However, no standard therapy currently exists after 1st line treatment for nmCRPC. Thus, an ARAT switch might prevent the progression of disease for those patients.

In conclusion, our study indicates the therapeutic potential of darolutamide as an ARAT switch for nmCRPC patients resistant to enzalutamide or apalutamide. Darolutamide, which has a different chemical structural formula, could be used as sequential therapy for nm CPRC patients following enzalutamide and apalutamide. This study was a small size, and further evaluation is a large‐scale study is necessary.

## AUTHOR CONTRIBUTIONS

Kazutoshi Fujita, Masahiro Nozawa, Kazuhiro Yoshimura, and Hirotsugu Uemura contributed to the study conception. Mamoru Hashimoto, Shogo Adomi, Ken Kuwahara, and Mamoru Hamaguchi collected the clinical data in our study. Takafumi Minami performed statistical analysis. Fujimoto S and Fujita K conducted the data analysis. Saizo Fujmoto wrote the manuscript. Each author gave final approval of the version to be published and agreed to be accountable for all aspects of the work.

## Funding information

The authors received no funding to support this work.

## CONFLICT OF INTEREST

Kazutoshi Fujita and Hirotsugu Uemura have honoraria from Janssen, Astellas and Bayer. Other authors have no conflict of interest to declare.

## ETHICS STATEMENT

The ethics committee of the Faculty of Medicine, Kindai University, approved the study protocol (approval number: R02‐247).

## Data Availability

Relevant data in this study could be obtained from the corresponding author by reasonable demand.
